# Proinflammatory macrophage-derived microvesicles exhibit tumor tropism dependent on CCL2/CCR2 signaling axis and promote drug delivery *via* SNARE-mediated membrane fusion

**DOI:** 10.7150/thno.45528

**Published:** 2020-05-17

**Authors:** Ling Guo, Ye Zhang, Runxiu Wei, Xiaochen Zhang, Cuifeng Wang, Min Feng

**Affiliations:** 1 School of Pharmaceutical Sciences, Sun Yat-sen University, University Town, Guangzhou, 510006, P.R. China; 2 Guangdong Provincial Key Laboratory of Chiral Molecule and Drug Discovery, School of Pharmaceutical Science, Sun Yat-sen University, Guangzhou, 510006, P.R. China

**Keywords:** microvesicles, proinflammatory macrophages, CCL2/CCR2 signaling, SNARE-mediated membrane fusion, metastatic ovarian cancer

## Abstract

**Background**: Exosome (Exo)-based chemotherapeutic drug delivery systems have been extensively investigated; however, the therapeutic potential of other subtypes of extracellular vesicles (EVs), in particular microvesicles (MiV), seem to be overlooked. Moreover, despite a general agreement on organ tropism of EVs, few studies have clearly demonstrated that EVs specifically target tumor tissue.

**Methods**: Proinflammatory macrophage-derived EV subpopulations comprising apoptotic bodies (ApB), MiV and Exo were isolated under differential ultracentrifugation, and further analyzed using comparative proteomic and lipid approach.

**Results**: On the basis of EV biogenesis pathways, our data demonstrated that MiV acquire the tumor-targeting capacity probably through inheritance of CCR2-enriched cell membrane which also drives the recruitment of donor cells to tumor sites. Further, our data validate MiV utilize SNARE-mediated membrane fusion to directly discharge doxorubicin to nucleus and bypass endocytic degradation.

**Conclusions**: Compared with other EV subtypes, MiV loaded with doxorubicin gain significant benefits in chemotherapeutic outcomes including survival rate improvements in metastatic ovarian cancer. Therefore, MiV represent a potent alterative to Exo and synthetic liposomes (Lipo) for tumor-targeting drug delivery.

## Introduction

EVs are cell-derived, membrane-bound nanovesicles used to exchange proteins, lipids and nucleic acids among cells 1. This intercellular communication enables donor cells to influence neighboring and distant recipient cells in a diverse range of normal biological processes and aberrant pathological processes 1,2. Most studies have highlighted that EVs possess an organ- or cell-specific tropism, and are capable of overcoming complex biological barriers to shuttle bioactive cargoes 3. On the basis of these advantages, EVs are likely to be developed as desirable natural drug carriers with low immunogenicity to enhance targeted delivery to action sites 4,5. In particular, some first-in-class chemotherapeutic drugs may benefit from EVs, since non-targeted free drugs are inefficient and have severe side effects. Nevertheless, there is a common consensus that the majority of EVs are rapidly cleared by specialized phagocytes in liver and spleen, but barely accumulate in tumor tissues after synthetic administration 6. In order to change this biodistribution, EVs may be modified with some targeting proteins or peptides originated from genetically engineered donor cells 7. Although this approach is likely promising, it seems not suitable for all cell types. To date, how to efficiently apply EVs with intrinsic targeting capabilities for a better therapeutic efficacy remains to be addressed.

EVs can be broadly classified into three main subsets: ApB, MiV and Exo depending on their distinctive biogenesis or release pathways 8. Exo are formed through the fusion of multivesicular bodies with plasma membrane. MiV directly bud from the plasma membrane, while ApB are released from the cells undergoing apoptosis. In comparison with Exo, ApB and MiV are more heterogeneous in size and can be larger ranging from nanometers to micrometers. However, it is difficult to distinguish different EV subtypes due to an overlap in vesicle size, shape and composition 9. Despite the fact that most studies have only focused on Exo, whether Exo really play a more prominent role in patho/physiological processes, or are conferred with higher diagnostic or therapeutic potential than other EV subtypes, appears to be debatable. According to the biogenesis mechanism, MiV are most likely to mirror the plasma membrane compositions whereas Exo membranes are loaded with a high density of endosomal membrane proteins. Thus, MiV may replicate cell surface markers to initiate certain signal transduction, and exhibit the similar target selectivity as donor cells.

Cell invasion and migration are the most common pathological features of advanced ovarian cancer and 75% diagnosed cases of which have poor survival rate 10,11. Most patients initially respond to the standard treatment of surgical debulking and chemotherapy, but over 70% of them develop drug resistance or relapse. Unlike dissemination through blood or lymph systems in other solid tumors, ovarian cancer cells can exfoliate from the ovary and colonize distant organs in the peritoneum, especially the omentum. Therefore, it remains a challenge to precisely track down and destroy migratory tumor cells confined to the abdominal cavity. Classically-activated macrophages, also termed as M1 macrophages, are highly differentiated and tend to recognize and capture tumor cells 12. Consistently, we have observed that doxorubicin (Dox)-loaded M1 macrophages are able to find out spreading ovarian cancer cells with the help of upregulated cell surface protein C-C chemokine receptor type 2 (CCR2) 13,14. However, as opposed to EVs, live cells are required to maintain cell activity and structural integrity to prevent off-target drug release 15. Moreover, cell therapy gives rise to the risk of cell engraftment and proliferation in the body. Therefore, EVs, particularly MiV, derived from M1 macrophages may inherit tumor-targeting capability, as well as avoid the risk of donor cells, so they could be developed as an alternative drug delivery system to seek and kill ovarian cancer cells. In this study, we provide experimental evidence to support this hypothesis.

## Materials and methods

### Materials

Doxorubicin hydrochloride (DoxHCl), chlorpromazine hydrochloride and amiloride hydrochloride were purchased from MedChem Express (USA). Filipin was obtained from Harveybio (Beijing, China). Botulinum toxin type A for injection was purchased from Lanzhou Institute of Biological Products (Lanzhou, China). Dox was prepared by desalting DoxHCl into the protonated form. RPMI-1640 medium, DMEM medium, fetal bovine serum (FBS), trypsin and penicillin/streptomycin were obtained from Gibco (Canada). TRIzol reagent and the far-red fluorescent, lipophilic carbocyanine DiD were the products of Invitrogen (USA). 4′, 6-Diamidino-2-phenylindole dihydrochloride (DAPI), dimethyl sulfoxide (DMSO), 3-(4,5-dimethylthiazol-2-yl)-2,5-diphenyl tetrazolium bromide (MTT), Lipopolysaccharides (LPS, L3024), lysosome isolation kit, bicinchoninic acid (BCA) protein assay kit and phospholipid assay kit were obtained from Sigma-Aldrich (USA). Interferon γ (IFN-γ, 315-05) was obtained from PeproTech (USA).

Total cholesterol assay kit was supplied by BioVision (USA). 2.5% glutaraldehyde and 4% paraformaldehyde solution were supplied by Leagene Biotechnology (Beijing, China). RNAiso plus, PrimeScript™ RT reagent kit with gDNA eraser (Perfect Real Time) and TB Green™ Premix Ex Taq™ II (TliRNaseH Plus) were purchased from Takara Bio (Shiga, Japan). RIPA lysis buffer, phenylmethanesulfonyl fluoride, primary and secondary antibody dilution buffer and loading buffer were purchased from Beyotime Biotechnology (Shanghai, China). Immobilon western chemiluminescent HRP substrate and polyvinylidene fluoride membranes were purchased from Millipore (USA). Chloroform-free lipid extraction kit, anti-thrombospondin-1 (TSP1) antibody (ab88529), anti-ADP-ribosylation factor 6 (ARF6) antibody (ab131261), anti-C-C chemokine receptor type 2 (CCR2) antibody (ab203128), anti-tumor susceptibility gene 101 (TSG101) antibody (ab30871), anti-apoptosis linked gene-2-interacting protein X (ALIX) antibody (ab88388) and anti-multidrug resistance protein 1 (MDR1, ABCB1) antibody (rabbit, ab129450) were supplied by Abcam (Britain). Anti-β Actin antibody (rabbit, 4970), anti-GAPDH antibody (rabbit, 8884), anti-rabbit IgG (7074) and the high-affinity F-actin probe Alexa Fluor 647 phalloidin were purchased from Cell Signaling Technology (USA). The commercial product of Lipo-Dox is a liposomal formulation resembling DOXIL® (Doxorubicin Hydrochloride Liposome Injection, manufactured by China Shijiazhuang Pharmaceutical Group Co., Ltd., Batch No: H20113320).

### Experimental cell lines and animals

A murine macrophage cell line RAW264.7, a human ovarian cancer cell line SKOV3 and a Chinese hamster ovary cell line CHO were purchased from the American Tissue Culture Collection. A stable green fluorescent protein (GFP)-expressing SKOV3 cell line was a gift from Qi Wang, Guangxi Medical University (Guangxi, China). All cells were incubated in a 37 °C humidified incubator (Thermo, USA) with 5% CO_2_. Female BALB/c nude mice (four-week old) were purchased from the Laboratory Animal Center, Sun Yat-sen University (Guangzhou, China) and kept under specific pathogen-free (SPF) conditions, with ready access to standardized food and water. All animal experiments were strictly observed under the Guiding Principles for the Use of Laboratory Animals approved by the Institutional Animal Care and Use Committee of the Sun Yat-sen University.

### Isolation of EVs

EVs-depleted medium was prepared by overnight ultracentrifugation at 120,000 g, 4 °C as described in reference 16. 2 × 10^8^ of RAW264.7 cells plated in a 150 mm dish were allowed to reach 80% confluence, and then stimulated upon exposure to recombinant murine IFN-γ (20 ng/mL) and LPS (0.5 μg/mL) for two days, followed by characterization of M1 macrophage specific markers. Afterwards, the medium was changed to a serum-free medium and the cells were allowed to grow for another 48 h. The conditioned medium was collected and the viability of the cells (> 95%) was confirmed with trypan blue exclusion test. EVs were harvested by sequential centrifugation as previously described 17 with some modifications. Briefly, conditioned medium was centrifuged at 500 g (Allegra X-22R with SX4250 rotor; Beckman) for 60 min at 4 °C to remove cells and cell debris. The supernatant was further centrifuged at 2,500 g (Allegra X-22R with SX4250 rotor; Beckman) for 60 min at 4 °C to pellet ApB. Subsequently, the supernatant was transferred to a new tube for another centrifugation step for 60 min at 20,000 g (Avanti J-E with F0850 rotor; Beckman) to collect MiV. Finally, Exo were obtained by the ultracentrifugation of supernatant from last step at 110,000 g (Optima L-90K with 90Ti rotor; Beckman) for 70 min. All pellets from each step were washed and resuspended in sterile PBS (150 μL).

### Isolation of endo-lysosomes and plasma membrane

The endo-lysosomes were isolated from M1 macrophages using an endo-lysosome isolation kit. Briefly, 2 × 10^8^ cells were pelleted by centrifugation at 600 g (Allegra X-30 with SX4400 rotor; Beckman) for 10 min. After homogenizing in Extraction Buffer on ice, the lysates were centrifuged at 1,000 g (Allegra X-30 with FX301.5 rotor; Beckman) for 10 min, followed by another centrifugation at 20,000 g for 20 min (Allegra X-30 with FX301.5 rotor; Beckman) to get crude endo-lysosome fractions. Then the samples were further purified by density gradient centrifugation (150,000 g for 4 h) on a multi-step OptiPrep gradient.

The plasma membranes of M1 macrophages were isolated as described previously 18,19. Briefly, 2 × 10^8^ cells were harvested and resuspended in ice-cold tris-magnesium buffer and extruded through a mini-extruder (Avestin, LF-1, Canada) for 20 times to disrupt the cells. The cell homogenate was mixed with sucrose to a final concentration of sucrose (0.25 M), and then was centrifuged at 2,000 g and 4 °C for 10 min. The supernatant was collected and further centrifuged at 3,000 g and 4 °C for 30 min to collect the cell membranes.

### Particle characterizations of EVs

Particle size of EVs was measured by Nanosight LM10 system (Malvern, UK) and qNano system (Izon Science, UK). All samples were diluted in PBS to a final volume of 1 mL. Ideal measurement concentrations were found by pretesting the ideal particle per frame value (20-100 particles/frame) on Nanosight instrument. The concentration of each sample was incrementally increased until the count rate reached a medium value at the highest laser attenuator value for tRPS measurements. For transmission electron microscope (TEM) imaging, the isolated EVs were mixed with paraformaldehyde at a final concentration of 4% wt/vol. After incubation at room temperature for 20 min, a drop of sample (100 µL) was added on a sheet of parafilm. A 300-mesh copper grid support film (Electron microscopy sciences, PA, USA) was placed on the sample liquid drop (membrane side down) to allow the membranes adsorption for 20 min. Next, the grid was washed three-times in a 100 µL drop of distilled water for 2 min, and then was transferred to a 100 µL drop of uranyl acetate solution for negative staining for 10 min. After washing away with unstained uranyl acetate, the grid was left to air-dry at room temperature. And the sample was observed under JEM1400 electron microscope (JEOL, Tokyo, Japan).

### Lipid extraction and quantification

Lipids in EVs, endo-lysosomes and plasma membranes were extracted using chloroform-free lipid extraction kit according to the manufacturer's protocol. Briefly, the lipids were extracted within one microliter of extraction buffer and dried overnight in a vacuum concentrator. Later, the dried lipids were resuspended by vigorous vortex in assay buffer (100 μL) for 5 minutes. Samples and standards are incubated for 30 minutes, and then were test with a standard 96-well fluorometric plate reader equipped with an excitation wavelength in the 530-570 nm range and an emission wavelength in the 590-600 nm range. Phospholipid levels are determined by comparison with known phospholipid standards. For cholesterol contents, samples are measured as compared to a known cholesterol standard curve within the 96-well microtiter plate. The phospholipid and cholesterol concentrations were normalized to the amounts of total protein.

### Loading and leakage profile of Dox in EVs

Dox (1 mM in DMSO) was allowed to incubate with EVs (20 mg/mL) for 24 h at room temperature. The product Dox unloaded in EVs was obtained by centrifugation at the speed of 300 g for 5 minutes. The precipitation was collected to trace the free Dox, and resuspended in 300 μL of DMSO and vortexed for 1 min to quantify the free Dox. The Dox concentration was determined by absorption at 481 nm using UV spectrophotometer (UV-2600, Shimadzu, Japan). A calibration curve was established using different concentrations of free Dox in phosphate buffer saline (PBS). Dox encapsulation efficiency (EE%) was calculated as follow: EE% = 1 - [mass of free Dox (mg)/mass of total Dox (mg)] × 100%.

To determine the leakage profile of three EV subsets loaded with Dox (EVs-Dox), a certain amount of EVs-Dox equivalent to 50 nmol of Dox was dispersed in 0.5 mL of DMEM containing 10% FBS and placed in 37 °C humidified incubator. At predetermined time intervals, the entire release medium was centrifugated at 300 g for 5 min. The released Dox was determined using UV spectrophotometer as described above. The leakage rate was calculated as a percentage of the released Dox from EVs-Dox against time.

### Cellular uptake test

SKOV3 cells were seeded at 150,000 cells per well in a 12-well plate for 24 h culture. EVs-Dox (equivalent to 4 μM of Dox) were added and further separately incubated for 2 h, 4 h or 24 h. Afterwards, the cells were harvested for FACS measurements (EPICS XL, Beckman, USA). The data are the mean fluorescent signals from 10,000 cells analyzed. For endocytosis inhibition analysis, cells were pretreated with clathrin-mediated endocytosis inhibitor chlorpromazine hydrochloride (CPZ, 28 μM), calveolar-mediated endocytosis inhibitor filipin (7.5 μM), or macropinocytosis inhibitor amiloride hydrochloride (50 μM) at 37 °C for 1 h, respectively. Then, EVs-Dox were added into the cells with or without inhibitors for 4 hour incubation at a total protein concentration of 80 μg/mL, which is equivalent to 4 μM of free Dox. As for Botulinum toxin serotype A (BONT/A) test, EVs-Dox were first pretreated with BONT/A overnight prior to adding to SKOV3 cells for further 4 h culture. All FACS results were analyzed using FlowJo software version 7.6.2. (Tritar Inc., San Carlos, California, USA).

### Cytotoxicity assay

Cells were incubated for 24 h at 5000 cells/well using 96-well plates (Costar, USA). When reaching to 80% confluence, the cells were exposed to varying concentrations of drugs for another 24 h. To determine cell viability, 20 μL of MTT solution (5 mg/mL, in phosphate buffered saline) was added to 100 μL of medium in each well of the 96-well plate. The plate was placed in a cell culture incubator until purple precipitates were clearly visible (approximately 4 h). Then, 150 μL of DMSO was added. The absorbance in each well was measured at 570 nm with a microplate spectrophotometer (Epoch, Bio-Tek, USA).

### Real-time cell invasion test

Cell invasion was monitored in a CIM-16 well plate by using xCELLigence real time cell analyzer (RTCA) instrument (ACEA Biosciences, San Diego, CA, USA). Each well was composed of an upper and a lower chamber separated by a microporous membrane containing randomly distributed 8 μm pores. A dimensionless parameter was measured as a cell-index which was used to evaluate the ionic environment at an electrode/solution interface and integrate information on cell numbers. As illustrated in Figure 3D, the complete medium (165 μL) was loaded in the lower well of the CIM-16 plate. Then the upper chamber was coated with matrigel (30 μL) and allowed to equilibrate at 37 °C for 4 h. Afterwards, cell suspension (10,000 cells in 100 μL) was seeded in the upper chamber, and then the whole plate was incubated for 30 min to allow the cells localizing on the membranes at room temperature in the laminar flow hood. After the addition of drug, each condition was performed in duplicate with a programmed signal detection schedule of every five minute during 48 h. All data were recorded by the supplied RTCA software (*vs.* 1.2.1). To analyze the results, cell index values of the selected wells at 1 h were set to a constant (Delta Constant) with a default value of one. After finishing the cell invasion tests in xCELLigence RTCA-DP instrument, the microporous membranes were fixed and imaging was done with a scanning electron microscope (S-3400N, Hitachi, Japan) at 10 kV. The acquired images were further edit using Adobe Photoshop CS6 (Adobe Systems Inc., USA).

### Western blotting analysis

Protein expression levels in cells and EVs were detected by denaturing, non-reducing SDS-PAGE electrophoresis. All cells or EVs were lysed in RIPA lysis buffer supplemented with protease inhibitor cocktail. The lysates were centrifuged at 15,000 rpm for 10 min at 4 °C and the supernatant was transferred to a new microtube for further detection. Total protein amount of cell extracts was measured using Pierce BCA protein assay kit (Thermo Scientific, USA). All samples (30 μg protein/lane) were run on 12% SDS-PAGE gels, and transferred to PVDF membranes (Millipore, USA). After blocking and incubation with primary and secondary antibodies, specific proteins were detected using ECL western blotting detection kit (GE Healthcare Bioscience, UK). Images were taken using Tanon 4600 (Tanon, China) and analyzed with software Image J (NIH, USA).

### RNA extraction and profiling

Total RNAs were extracted from SKOV3 cells treated with EVs-Dox, Lipo-Dox and DoxHCl (equivalent to 4 μM of Dox) using TRIzol (Invitrogen, USA), and futher were purified through the RNeasy Mini Kit (Qiagen, USA). RNA concentration was determined using a NanoDrop 2000 spectrophotometer (ND-2000, Thermo Fisher Scientific, Waltham, USA). cDNA was generated from purified RNA (1 μg) using the iScript cDNA Synthesis Kit (Bio-Rad). TaKaRa Taq was used for the PCR. The primers were used as follows: MDR-1: CAGGAACCTGTATTGTTTGCCACCAC (for), TGCTTCTGCCCACCACTCAACTG (rev); CCL2: AGAATCACCAGCAGCAAGTGTCC (for), TTGCTTGTCCAGGTGGTCCATG (rev). RNA integrity was determined by an Agilent 2100 Bioanalyzer (Agilent Technologies, Palo Alto, USA).

### RNAi downregulation assay

SKOV3 cells were transfected using Lipofectamine RNAiMAX transfection reagent (Life Technologies, Germany) according to the supplier's instructions. Briefly, SKOV3 cells were seeded in 12-well plates at a density of 3.75 × 10^5^ cells/well in RPMI 1640 culture medium (with 10% heat-inactivated FBS and without penicillin and streptomycin) 24 h prior to transfection. After removal of medium, siMDR1 complexes were prepared at a final concentration of 50 nM in 0.5 mL of Opti-MEM, and then added into each well. Scrambled siRNA was used as a control. At 24 h post-transfection, SKOV3 cells were challenged to EVs-Dox at a Dox concentration of 4 μM for another 24 h. Western blotting analysis was used to evaluate the P-glycoprotein (P-gp) downregulation levels. Anti-MDR1 small RNA sequences were used as followed: 5′-GGAUAUUAGGACCAUAAAUtt-3′ (sense), 5'-AUUUAUGGUCCUAAUAUCCtg-3' (antisense).

### Mass spectrometric analysis

Total EVs or their donor cells were homogenized in 1 mL RIPA lysis buffer supplemented with cocktail (1 mM), and lysed on ice for 30 min. The homogenate was then centrifuged at 10,000 g for 20 min at 4 °C according to the method described previously with some modifications 20,21. The supernatant was diluted with ammonium bicarbonate buffer (50 mM) to a final concentration of 3 μg/μL. The proteins in the solution were allowed to precipitate out overnight at -20 °C by the addition of precooled acetone 4:1 (v/v), and then centrifuged at 15,000 g for 30 min at 4 °C. The pellet was successively washed with precooled acetone, 70% ethanol, and acetone seriatim. The solvent was removed by centrifugation at 15,000 g for 10 min at 4 °C each time. Finally, the samples were immediately frozen and lyophilized. The lyophilizate was dissolved in urea buffer (50 μL). Then, 50 mM DTT (2 μL) was added. The mixture was incubated at 56 °C for 30 min, and subsequently was cooled at 30 °C. Then iodoracetamide (IAA) was added at a final concentration of 10 mM in the dark and incubated for 30 min at 30 °C. The excess IAA was quenched by the addition of DTT for 30 min. The sample was left to digest overnight at 37 °C by the addition of trypsin at an enzyme to substrate weight ratio of 1:50. Finally, the samples were acidified with 0.6% trifluoroacetic acid (v/v) to stop the digestion process according to the method described previously with some modifications 22,23. The protein concentration was quantified using the BCA kit. The peptide samples were desalted and concentrated using C18 tips (87782, Thermo Fisher Scientific Inc., Waltham, MA), and then lyophilized.

The shotgun assay was performed on a nanoRPLC-Q Exactive Orbitrap mass spectrometer (Thermo, CA, USA). The peptide samples in 0.1% formic acid (v/v) were separated using a C18 column (75 μm × 150 mm, NanoViper C18, 2 μm, 100 Å; Thermo, PA, USA). Eluents A and B were 0.1% formic acid in water and 0.1% formic acid in 80% acetonitrile, respectively. The elution gradient was set as follows: 0 ~ 95 min, 3%→32% (B); 95 ~ 105 min, 32%→100% (B); and 105~120 min, 100% (B) for a total run time of 120 min at a flow rate of 300 nL/min.

All MS and MS2 spectra were collected with the 10 most intense ions fragmented by collision-induced dissociation. Full mass scan was acquired in the range of 355 ~ 1700 m/z with a mass resolution of 70,000.

### Biodistribution studies

Purified EVs were fluorescently labeled using Vybrant® DiD according to manufacturer's instructions with some modifications. Briefly, EVs and Lipo were incubated for 10 minutes with DiD (5 μM), and then washed with PBS (1 mL). A xenograft murine model of human epithelial ovarian cancer was established by injection of 1 × 10^7^ SKOV3 cells in 200 µL of PBS/Matrigel solution (50%, v/v) into the abdomen of the BALB/c nude mice. After two weeks, tumor-bearing mice and healthy mice were intraperitoneal (*i.p.*) injected with various EVs and Lipo labeled by DiD. The mice were sacrificed 24 h post-injection. Tumors and major organs were collected and imaged by using the NightOWLII LB983 (Berthold Technologies, Bad Wildbad, Germany). All images were acquired with an exposure time of 5 seconds, and further analyzed with indiGO software (Berthold Technologies, Bad Wildbad, Germany).

### *In vivo* therapeutic efficacy

After establishment of metastatic ovarian cancer xenograft murine model at day 7 and 12, magnetic resonance imaging (MRI) was used to assess tumor progression. Mice were pretreated with 1.2% isoflurane inhalational anesthesia. All MRI was conducted using the M3^™^, a compact, high-performance MRI system (Aspect Imaging, Israel), equipped with a 30 mm inner diameter transmit/receive volume radio frequency coil. Coronal abdominal images of each mouse before and after tumor cells administration were obtained. T2 weighted images were acquired using Fast Spin echo sequence with TR = 3500 ms, TE = 53 ms, FOV = 30 × 30 mm, slice thickness = 1 mm and matrix = 196 × 196. T1 weighted images were acquired using Spin Echo sequence with TR = 400 ms, TE = 12 ms, FOV = 80 × 30 mm, slice thickness = 1 mm and matrix = 256 × 96. Total acquisition time was 19 min.

At stage II ovarian cancer, all mice were randomly assigned to 6 groups (n = 6 per group). Various samples containing equivalent Dox concentration of 2 mg/kg were given to mice by *i.p.* injection every 3 days for 3 weeks. At stage III ovarian cancer, the mice were treated various samples as the same doses as stage II, but every 2 days for 20 days. At day 28, the mice were sacrificed and their organs were harvested, photographed and weighed. Then, major organs were subjected to histopathological examination after fixed in 10% neutral formalin and desiccated and embedded in paraffin. SKOV3/GFP tumor growth was monitored every week using the NightOWLII LB983 (Berthold Technologies). Maximum/minimum of the fluorescence signal intensity was adjusted to be the same throughout the experiment.

### Statistical analysis

All statistical analysis was performed using Prism 7.0 software (GraphPad Software) by an unpaired Student's t-test, one-way or two-way ANOVA with Bonferroni multiple comparisons post-test. Statistical significance for survival curve was calculated by the log-rank test. Data were approximately normally distributed and variance was similar between the groups. Statistical significance is indicated as * p < 0.05, ** p < 0.01, *** p < 0.001, and **** p < 0.0001.

## Results

### Proinflammatory macrophage-shed MiV underwent a tumor-specific tropism contributed by inheritance of CCR2-enriched cell membrane

**Figure 1:**
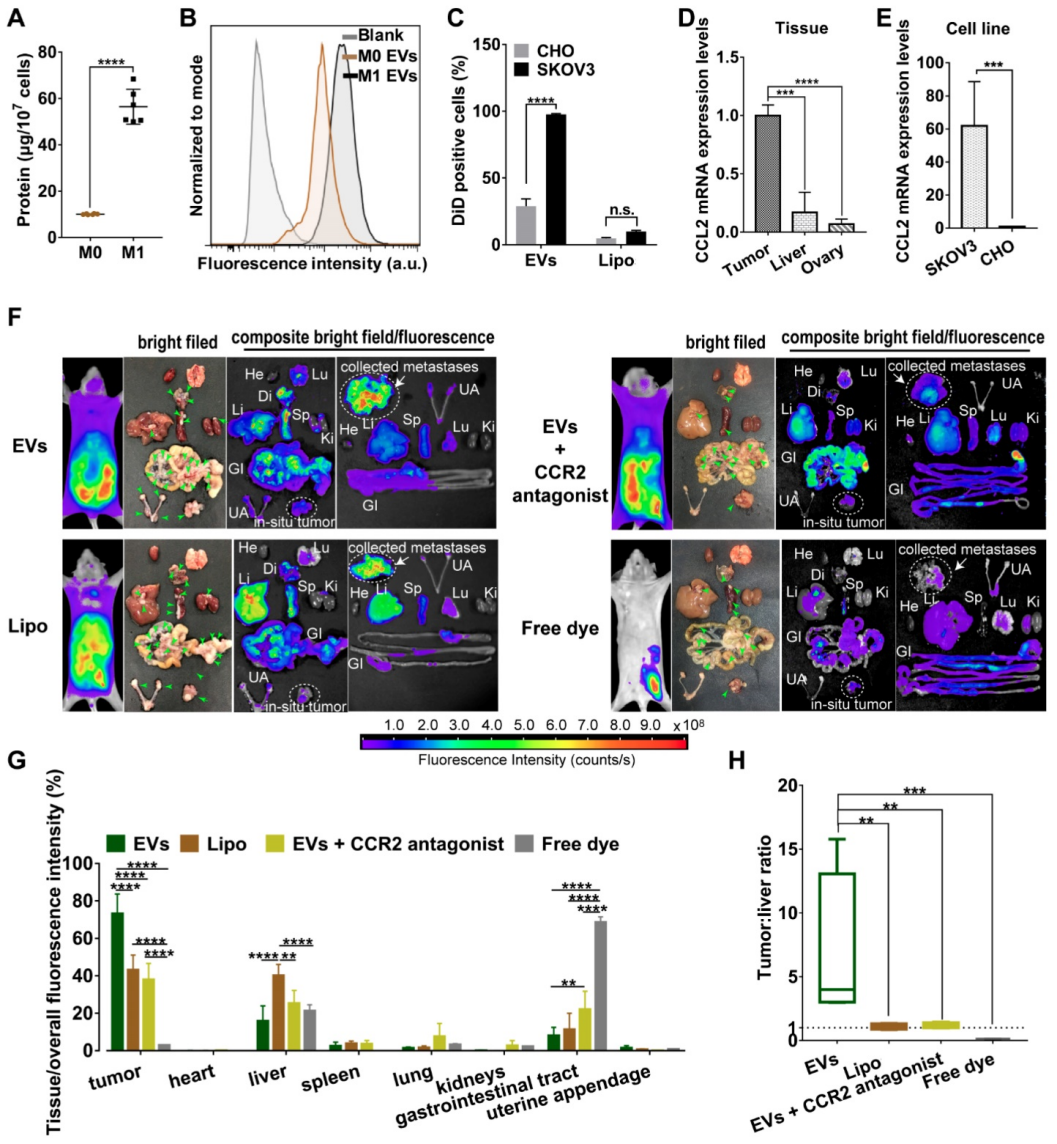
M1 macrophage-derived EVs displayed a tropism for tumor cells. (A) Total protein contents of EVs produced by M0 macrophages and M1 macrophages, normalized per ten million cells (n = 6). (B) The representative fluorescence intensities of DiD-positive cells at 4 h were displayed. (C) Cellular uptake efficiency of CHO cells or SKOV3 cells after incubation with EVs or Lipo labeled with DiD was measured by flow cytometry at 4 h. (D) CCL2 mRNA expression levels in SKOV3 cells and CHO cells. (n = 6). (E) CCL2 mRNA expression in tumor nodules, liver and ovary (n =6). (F) Bright-field and composite bright field/fluorescence images of excised organs from mouse models of metastatic ovarian cancer at 24 h post-injection of DiD-labeled EVs, Lipo or free DiD with the same molar amount of DiD (amount 5 nmol per mouse, n = 3). For CCR2 antagonist experiments, INCB3344 was administered to mice by oral gavage for 12 hours later prior to injection of EVs. Green arrow heads indicate the metastatic nodules. Color bars show the low- and high-intensity values in units of counts/s. (G) Biodistribution profiles of each experimental group at 24 h measured by fluorescence imaging in the heart (He), liver (Li), spleen (Sp), lung (Lu), kidneys (Ki), gastrointestinal tract (GI), uterine appendages (UA) and tumors. (H) Bar graph showed calculated tumor to liver tissue uptake ratios. The data were shown as mean ± s.d., ** = p < 0.01, *** was p < 0.001, **** was p < 0.0001 by Student's t test, one-way ANOVA test or two-way ANOVA test.

Compared with non-polarized M0 macrophages, pro-inflammatory phenotype M1 macrophages overexpressed surface chemokine receptors *i.e.* CCR2, leading to a strong recruitment to the site of tumor though chemotactic attraction 13. Likewise, topology of EVs embodied that extracellular receptors and ligands were positioned on the outside membrane, while cytoplasmic proteins and RNAs were enclosed inside. Since all EV compositions originate from secreting cells 24, M1 macrophage-released EVs may inherit specific cell targeting properties from donor cells. To prove this hypothesis, we first collected all EVs from conditioned medium of M1-polarized macrophages whereby RAW264.7 cells were pre-activated by LPS and IFN-γ for 48 h. Exogenous stimuli could promote cells to release EVs 25. Compared with steady-state M0 macrophages, equal numbers of M1 macrophages indeed released more EVs with a higher protein yield showing a better tumor-selective uptake rate (Figure 1A-1C). By contrast, there was no significant difference in internalization of Lipo between cancer cells and normal cells. Furthermore, EVs were labeled with fluorophore DiD and *i.p.* administered into orthotopic ovarian cancer mice models (4 mg total protein/kg). It was observed that EVs mainly retained in metastatic nodules (green arrow heads) and much less in the liver, while negligible fluorescent signals were detected in other organs including heart, lung, spleen, kidneys, gastrointestinal tract, and uterine appendage (Figure 1F-1G). It was unlikely that fluorescent dye leaked from EVs, since free DiD was rapidly cleared from the circulation as a result of extremely low signals. By contrast, fluorescent of Lipo accumulated in tumor were 24.0-30.1% lower than that of EVs containing the same amount of DiD. In addition, Lipo seemed to be equally distributed in liver and tumor tissues with a fluorescence ratio of 0.93, which suggested that Lipo lacked tumor specificity after *i.p.* administration (Figure 1H). According to the elevated expression of chemokine CCL2 in cancer cells and tumor tissues (Figure 1D-1E), we speculate CCR2 may correlate with tumor tropism of EVs as their donor cells. Next, a small molecule CCR2 antagonist of INCB3344 was employed, and consequently a significant reduction of fluorescent signals was observed in the presence of INCB3344 (Figure 1F-1G). Collectively, EVs originated from M1-polarized cells represented as a promising platform to specifically transfer the therapeutic cargo to tumor tissue; however, it was still unclear which subtype of EVs was associated with the CCR2-dominated tumor selectivity.

**Figure 2:**
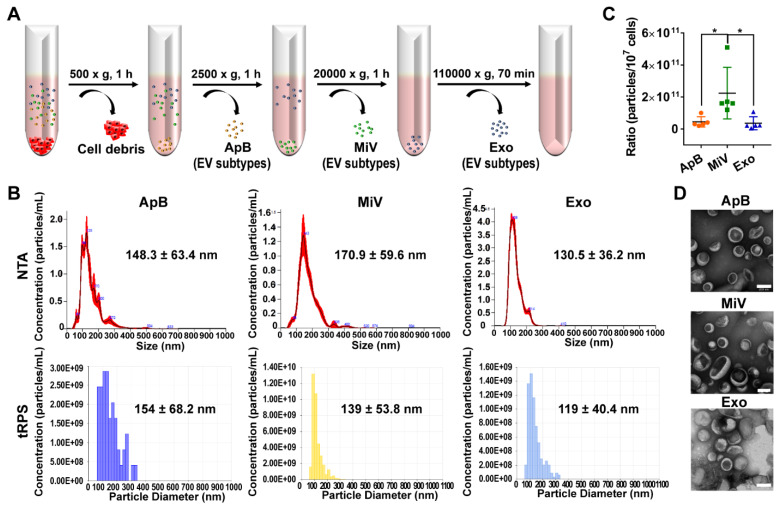
Heterogeneous populations of EVs with overlapping sizes and similar appearance. (A) Schematic depiction of the isolation protocol for ApB, MiV, and Exo. (B) Size distribution of EV subtypes was determined by NTA and tRPS, respectively (*n* = 3). (C) Numbers of EV subtypes secreted by 10^7^ M1 macrophages were measured by NTA (*n* = 5). (D) TEM images of isolated ApB, MiV, and Exo. Scale bars are 200 nm. The data are shown as mean ± s.d., * = p < 0.05 by one-way ANOVA test.

**Figure 3:**
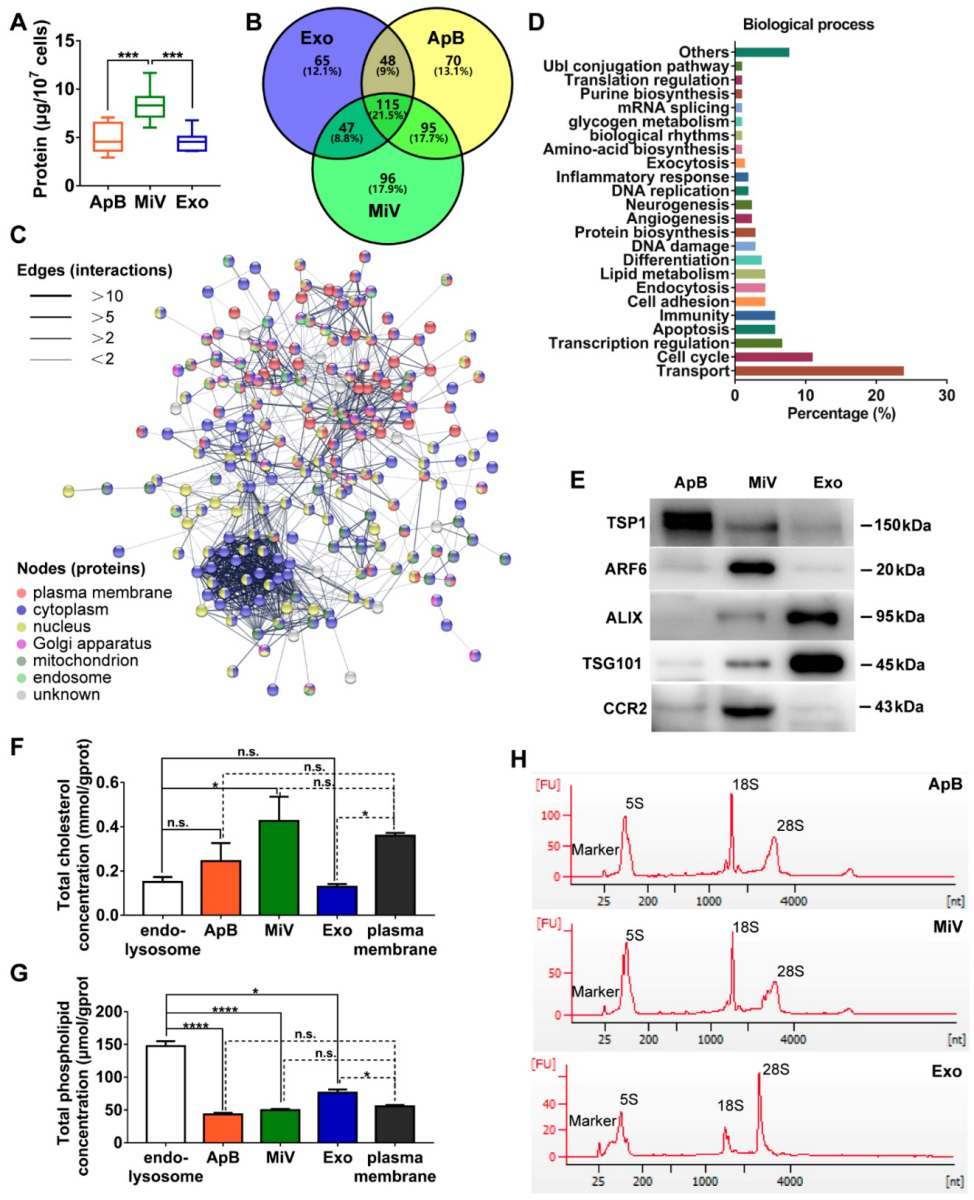
Distinct protein, lipid and RNA profiles of EV subtypes. (A) Total protein contents of ApB, MiV, and Exo produced by ten million of M1 macrophages were measured by BCA (n = 5). (B) Venn diagram shows the number of overlapping genes across pairwise comparisons of proteins whose expression levels in EV subtypes similar with donor cells. Venn diagram is created using Venny 2.0. (C) Connectivity diagrams summarizing proteomic analysis of common proteins between MiV and donor cells associated with subcellular localization using the String online dataset. Thickness of edges and nodes of different colors represent the predicted functional associations and distinct compartments. (D) Functional classification of common proteins between MiV and donor cells from Gene Ontology annotation. Histograms represent the assigned classification of biological process. (E) EV markers of TSP-1, ARF6, TSG101, ALIX and M1 macrophage surface marker CCR2 were detected by western blotting analysis (n = 3). (F) Total cholesterol levels and (G) total phospholipid levels in EV subtypes, endo-lysosomes and plasma membrane (n = 3). (H) Electropherogram depicting total RNA pattern from EV subsets was analyzed by Agilent 2100 BioAnalyzer. The data are shown as mean ± s.d., * = p < 0.05, *** = p < 0.001, **** = p < 0.0001, n.s. = p > 0.05 by one-way ANOVA test.

Different types of EVs comprising ApB, MiV and Exo were further purified under differential ultracentrifugation steps (Figure 2A). According to the standard EV isolation method 16, we increased the low-speed spinning rate and time to remove the cell debris and larger EV pellets which are unsuitable for drug delivery. Then, ApB, MiV and Exo were subsequently collected without further optimization. EV size and concentration were first assessed by nanoparticle track analysis (NTA, Nanosight^TM^) and tunable resistive pulse sensing method (tRPS, qNano), respectively. Both NTA and tRPS measurements revealed that Exo seemed to be the smallest particle population (119 ~ 130.5 nm) with a narrow distribution, while ApB and MiV were marginally larger and more heterogeneous in size (ranging from 60 to 400 nm, Figure 2B). Although the three subtypes were comparable in particle size, MiV were the largest population secreted from M1 cells with a concentration of approx. 2.24 × 10^11^ particles every ten million cells, which was significantly higher than ApB and Exo (Figure 2C). Additionally, similar morphology of the three different vesicles was observed by TEM, showing cup-like concavity structure with a diameter of ~ 200 nm (Figure 2D). Finally, we tried to discern EV subpopulations based on their protein, lipid and RNA contents. MiV harbored more total proteins than the other subtypes, which might be due to the high-yield isolation from macrophages (Figure 3A). Then, the proteome of all EV subtypes was further examined by liquid chromatography-tandem mass spectrometry (LC-MS/MS). We selected proteins whose expression levels in EVs were similar to donor cells by setting a mean fold change of ≤ 1.0 as an arbitrary threshold. Although many proteins were shared by three EV subpopulations, MiV displayed the highest degree of similarity with donor cells (Figure 3B). Among these common proteins between MiV and donor cells, we further analyzed the subcellular localization of their interacting proteins, predicting an enrichment of interologs present in nucleus/cytoplasma and cytoplasm/plasma membrane (Figure 3C). Furthermore, most of the proteins were involved in transport process catalogued by Gene Ontology (GO) analysis (Figure 3D). In terms of EV specific markers, western blotting analysis was performed and identified that ApB expressed apoptotic body-specific surface marker TSP-1, while MiV highly enriched with microvesicle marker ARF6 (Figure 3E and Supplementary Figure S1) 26. Moreover, Alix and TSG101 (generally used as exosomal markers 27) were barely expressed in ApB and MiV, suggesting the obtained three EV subgroups under our established protocol were purified. Strikingly, CCR2 expression of MiV was markedly higher than that of other types, which suggested MiV were more likely to shed from cell plasma membrane, since CCR2 was mainly confined to cell surface 28. In addition, we also observed that MiV and ApB have high levels of cholesterol and low levels of phospholipids, which is similar with plasma membrane (Figure 3F and 3G). On the other hand, Exo originated from both endocytic membrane and plasma membrane; therefore, its lipid compositions were in the middle of these two kinds of cellular membrane 29. In addition to different protein and lipid profiles, we further assessed the total RNA enclosed in each EV type using Agilent 2100 Bioanalyzer (Figure 3H). All samples were mainly mapped to ribosomal RNA sequences including 28S, 18S and 5S subunits. In particular, higher 28S/18S ratio verified that Exo samples contained purified RNA of high quality. Together, MiV seemed to be the largest EV subpopulation which could be hardly distinguished from ApB and Exo by sizing and appearance according to our established protocol; however, various bioactive contents demonstrated that the three subsets were derived from distinct biogenesis and release pathways. Whether ApB and Exo possessed tumor selectivity remained to be elucidate, although our current data strengthened that MiV directly budded and pinched off from the cell membrane and significantly enriched with CCR2, which allowed MiV to recognize tumor cells in response to chemokine gradient.

### MiV enhanced Dox-induced cell death with improvement of chemosensitivity and inhibition to cell invasion

**Figure 4:**
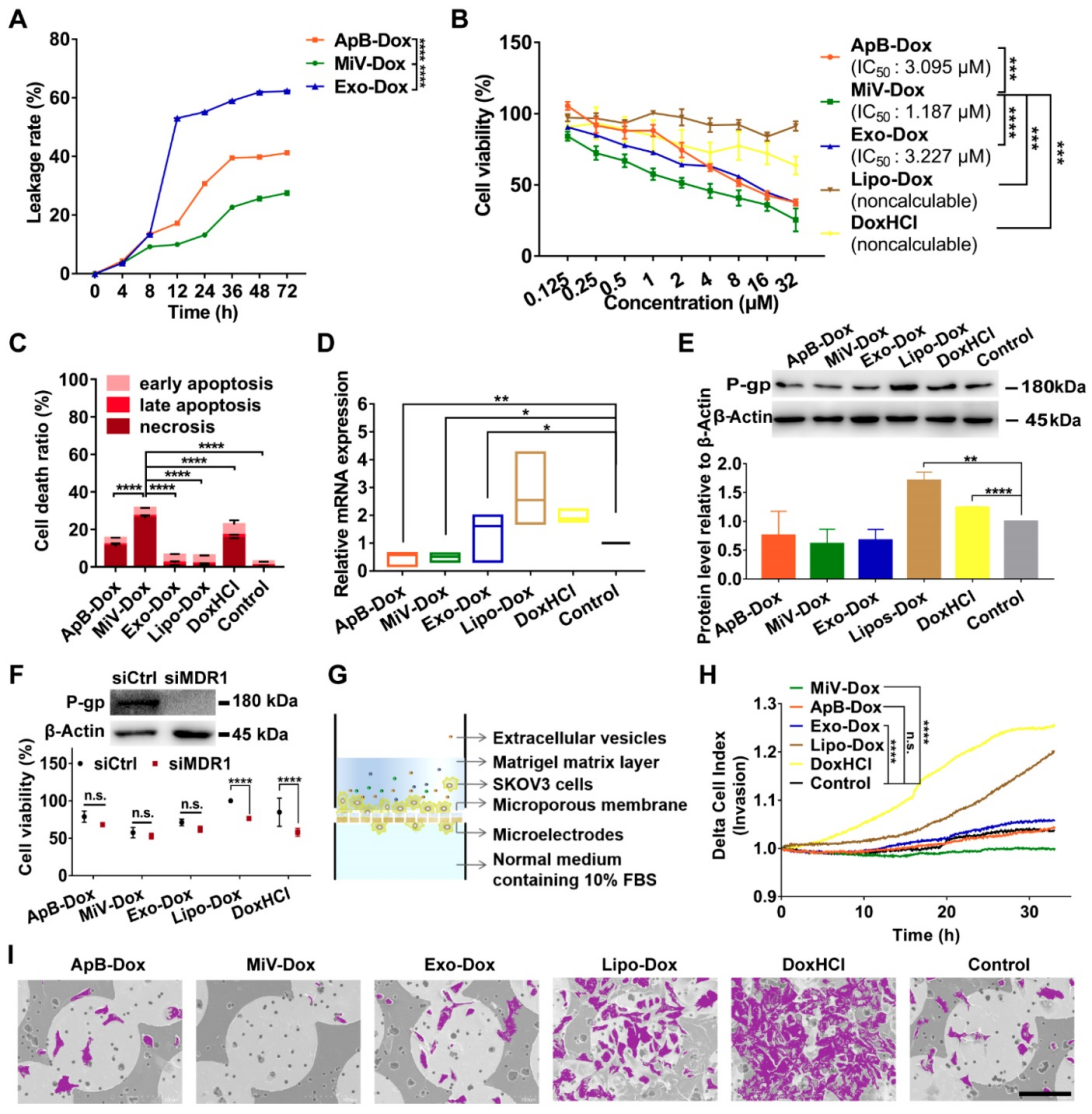
MiV loaded with Dox exerted an optimal anticancer effect without induction of drug resistance while preventing cell invasion. (A) *In vitro* Dox leakage from EVs in cell culture medium at 37 °C during 72 h (n = 3). (B) Dose-response curves for cell viability of SKOV3 cells with treatment of ApB-Dox, MiV-Dox, Exo-Dox, Lipo-Dox and DoxHCl for 24 h. (C) Annexin V-FITC/PI assay for apoptosis detection of SKOV3 cells under the treatment of ApB-Dox, MiV-Dox, Exo-Dox, Lipo-Dox and DoxHCl (equivalent to 1.187 µM Dox which is the IC_50_ value of MiV-Dox against SKOV3 cells) for 24 h. (D) The mRNA levels of MDR1 were detected by real-time PCR analysis, and normalized to the control GAPDH mRNA levels. (E) Representative western blot images and quantification of protein levels in SKOV3 cells after exposure to various formulations. An equivalent amount of total protein was analyzed with antibodies against P-gp, and β-Actin was used as the loading control. (F) SKOV3 cells were pretreated with MDR1 siRNA overnight prior to incubation with each formulation containing the same amount of Dox for 24 h. Then cell viability assay was further performed to evaluate the impacts of MDR1 downregulation on chemotherapy. Down-regulation of P-gp verified by western blot analysis. An equivalent amount of total protein was analyzed with antibodies against P-gp, and β-Actin was used as the loading control. (G) Schematic illustration of real-time cell invasion assay. (H) Invasion rate of SKOV3 cells induced by Dox-loading formulations (equivalent to 2.374 μM Dox) was monitored by a real-time cell analyzer. (I) Representative SEM images of the migratory SKOV3 cells grown on microporous membranes. Cells were colored purple using Adobe Photoshop CS6. Scale bar is 100 µm. All experiments were conducted in at least three independent replicates. The data are shown as mean ± s.d., * = p < 0.05, ** = p < 0.01, *** = p < 0.001, **** = p < 0.0001, n.s. = p > 0.05 by one-way or two-way ANOVA test.

In order to clarify whether M1 macrophage-derived EVs were competent for chemotherapeutic drug delivery, Dox was chosen as a model drug and loaded with each type of EVs (named as ApB-Dox, MiV-Dox and Exo-Dox) by incubation at room temperature for 24 h. The drug entrapment efficiency of each EV was measured and higher than 98%, implicating a desirable drug-payload capacity (Table S1). However, Exo-Dox showed the highest degree of leakage, probably due to Exo membrane destabilized with relatively low levels of cholesterol 30. By contrast, Dox was monitored to leak from ApB-Dox and MiV-Dox at a slow rate (approx. 15% in 12 h) (Figure 4A). Moreover, MiV-Dox was capable of enclosing more than 86% of Dox during 24 h incubation, which guaranteed minimal drug leakage during transport. Then, anti-tumor effects of EVs-Dox were further evaluated by assaying cell viability and apoptosis in SKOV3 cells. In comparison with free drug DoxHCl and commercial liposome formulation Lipo-Dox, all EVs-Dox showed pronounced inhibitory effects on tumor cells growth with IC_50_ values in the range of 1.187 μM ~ 3.227 μM (Figure 4B and Figure S2). Surprisingly, though there was no different cytotoxicity observed among each EV vesicle on either tumor cells or normal cells (Figure S3 and S4), MiV loaded with Dox appeared to be more toxic to cancer cells than ApB-Dox and Exo-Dox, which was also evidenced by apoptotic cells tests (Figure 4C).

Drug resistance and tumor metastasis/invasion are two major obstacles in ovarian cancer chemotherapy. We then tested MDR1 mRNA and protein expression levels, since tumor cells could enhance the drug efflux mediated by MDR1 to circumvent the cytotoxic action. DoxHCl as well as Lipo-Dox significantly induced MDR1 upregulation leading to a decreased cellular drug accumulation (Figure 4D and 4E). Interestingly, no alterations in MDR1 expression were observed in the cells prior to and after treating with all EVs-Dox. It was further confirmed that all EVs-Dox possessed cytotoxicity independent of MDR1; however, knockdown of MDR1 sensitized tumor cells to DoxHCl and Lipo-Dox (Figure 4F). Additionally, Dox-induced migration and invasive behavior of SKOV3 cells upon exposure to various formulations were further monitored using RTCA (Figure 4G). As expected, free drug DoxHCl remarkably promoted SKOV3 cell invasion compared with drug-encapsulated in Lipo. Interestingly, tumor cell invasion was completely inhibited by MiV-Dox; however, similar migration profiles were tracked in the cells treated with other EVs-Dox or without any treatment (Figure 4H). Consistently, different amounts of migrated cells across microporous membranes of RTCA system were also observed by scanning electron microscope (SEM) technique (Figure 4I and Figure S5). Overall, these results indicated that M1 macrophage-derived MiV, compared with the two other subsets ApB and Exo, not only enhanced the cytotoxicity of Dox, but also significantly inhibited Dox-elicited tumor cell migration without induction of drug resistance.

### MiV utilized SNARE-mediated membrane fusion machinery for intracellular delivery of Dox

**Figure 5:**
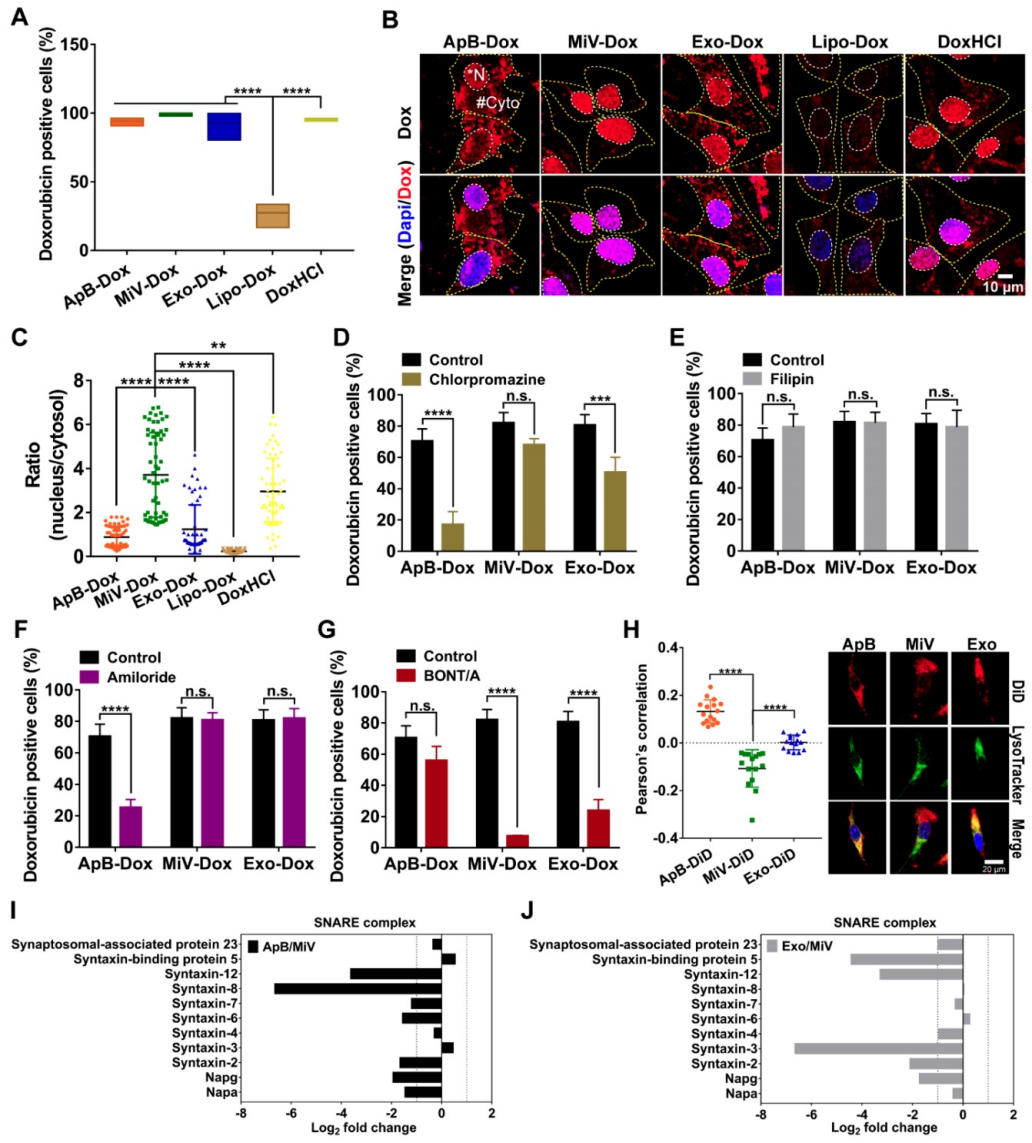
SNARE-mediated membrane fusion pathway facilitated intracellular delivery of Dox by MiV. SKOV3 cells were incubated with Dox-containing formulations (equivalent to 4 µM of Dox) prior to further measurements. (A) Percentages of positive cells was determined by flow cytometry at 4 h. (B) Confocal laser scanning microscopy images showed the intracellular distribution of Dox of SKOV3 cells after treated with various formulations at 4 h. The nuclei were stained with DAPI (blue). Dox produced the red fluorescence. The merged images were the overlay of two individual images. White dotted line marked the region of nucleus (*N). Yellow dotted line indicated cytoplasm (#Cyto). Scale bar was 10 µm. (C) The ratio of nuclear to cytoplasmic Dox fluorescence was quantified based on quantification of randomly selected cells (n = 60) with Image J. (D-G) The Dox-positive cells were analyzed by flow cytometry. SKOV3 cells were pretreated with (D) chlorpromazine (28 μM), (E) Filipin (7.5 μM) or (E) amiloride (50 μM) for 1 h before addition of ApB-Dox, MiV-Dox and Exo-Dox (equivalent to 4 µM of Dox) for another 4 h. (G) ApB-Dox, MiV-Dox and Exo-Dox were pretreated with BONT/A overnight before being added into SKOV3 cells for further quantification. (H) A colocalization study using LysoTracker Green as a standard endo-lysosomal marker. Representative images of SKOV3 cells stained with LysoTracker (green) and DAPI (blue) after being incubated with ApB-Dox, MiV-Dox and Exo-Dox (red) for 4 h. Scale bar was 20 μm. Pearson's correlation coefficient for Dox co-localized with endo-lysosomes was calculated using ImageJ software (n = 15 cells). (I-J) Proteomics analysis of three EV subsets by LC-MS/MS. Bar graph shows the changes of SNARE proteins in ApB and Exo compared to MiV. The data are shown as mean ± s.d., ** = p < 0.01, *** = p < 0.001, **** = p < 0.0001, n.s. = p > 0.05 by one-way or two-way ANOVA test.

In attempt to understand how MiV could optimally achieve maximal cell-killing effects, we first evaluated the cellular uptake ability of all formulations using SKVO3 cells. FACS data showed that EVs-Dox as well as DoxHCl alone were rapidly internalized into tumor cells, and the percentages of drug-positive cells were nearly 100% after 4 h of incubation. By contrast, internalization of Lipo-Dox proceeded much more slowly, achieving only 27% of positive cells (Figure 5A). This might explain the reduced anti-tumor effect of Lipo-Dox compared to either free drug or EVs-Dox (Figure 4B and 4C). In agreement with FACS data, Lipo-Dox displayed considerably weak Dox fluorescence after incubation with SKVO3 cells for 4 h. Additionally, EVs loaded with Dox showed differing intracellular distribution. ApB-Dox and Exo-Dox showed a number of punitive fluorescent signals in cytosol, whereas Dox was rapidly released from MiV and trafficked into nucleus where cell-killing was initiated (Figure 5B, C and Figure S6, S7). Surprisingly, DoxHCl showed a similar intracellular distribution pattern as MiV-Dox. Nevertheless, DoxHCl did not achieve the comparable antitumor effect. It was probably due to the fact that a large amount of Dox was pumped out from tumor cells with elevated P-gp expression 31.

Next, uptake kinetics was further investigated in an effort to understand the difference in antitumor efficacy among the three subsets of EVs-Dox, since the route of EV internalization might dictate the functional response or efficiency of cargo delivery. A heterogeneous population of EVs were taken up into recipient cells *via* a variety of routes, mainly including clathrin-mediated endocytosis, caveolin-mediated endocytosis, macropinocytosis and plasma or endosomal membrane fusion 32. To test the possibilities in EVs-Dox uptake pathways, a range of inhibitors were employed. Cell entry of EVs-Dox appeared not to be affected by filipin as an inhibitor of caveolae-mediated routing. However, ApB-Dox intake was significantly inhibited by the addition of chlorpromazine and amiloride, implying ApB-Dox might enter into a cell through clathrin-mediated endocytosis and macropinocytosis (Figure 5D-5G). Of note, the percentage of Dox-positive cells was remarkably decreased by 74.55% under BONT/A treatment (which is used to impair SNARE-mediated membrane fusion) 33. By contrast, a reduction ranging from 30.23% to 56.78% in Exo-Dox uptake efficiency upon chlorpromazine or BONT/A suggested that Exo-Dox could be internalized either through clathrin-mediated endocytosis or membrane fusion. Moreover, all detected SNARE proteins by proteomic analysis exhibited higher abundance in MiV samples compared with either ApB or Exo (Figure 5I and 5J), which might be involved in assisting and regulating membrane fusion process. Additionally, the extent of colocalization of MiV and endo-lysosomes was significantly lower than that of ApB and Exo (Figure 5H). Thereby, MiV-Dox might undergo fusion with target cells to avoid endocytic degradation and rapidly discharge cargo to nuclei 34.

### MiV with Dox payload exerted robust antitumor efficacy in advanced ovarian cancer models

**Figure 6:**
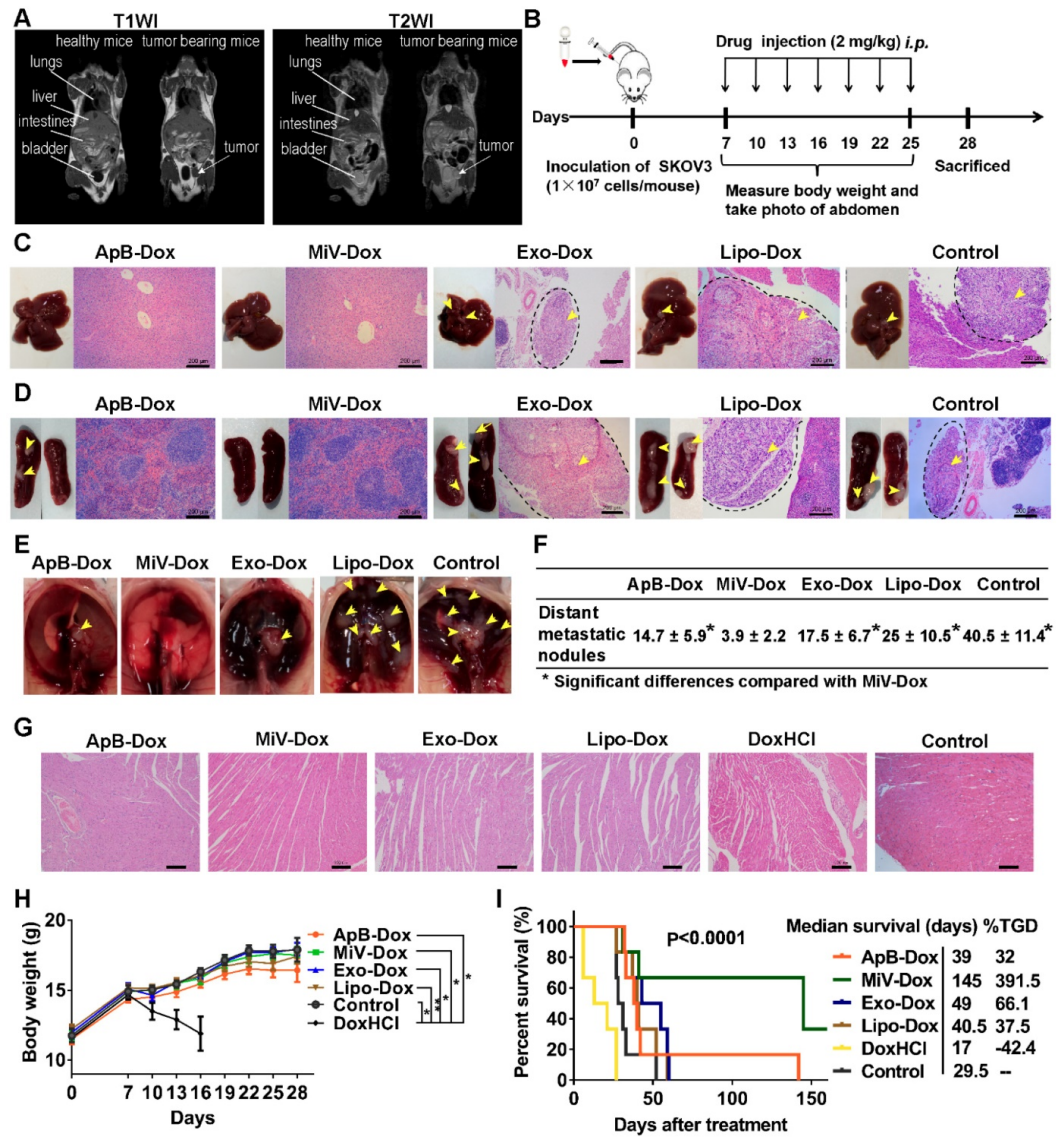
*In vivo* anti-metastatic efficacy of EVs-Dox against stage II ovarian carcinoma. (A) T1-weighted images (T1WI) and T2-weighted images (T2WI) of healthy mouse and tumor-bearing mouse in the coronal plane. Arrows indicate metastatic regions where the signals were hypointense as compared with adjacent normal tissues in T1WI while hyperintense in T2WI. (B) Experimental procedure of tumor induction and therapeutic regimen. (C-E) The location of metastatic nodules on livers, spleens and diaphragms in control and treated mice (n = 8 per group). Tumor nodules are indicated by arrow heads. Scale bars are 200 µm. (F) The number of metastatic nodules was counted in control and Dox-treated mice (n = 8 per group). (G) H&E staining images of heart tissue from tumor-bearing mice with or without any treatments on day 28. Scale bars are 100 µm. (H) Whole body weights after different treatments during 28 days (n = 8 per group). (I) Kaplan-Meier survival curves and the median survival time of tumor-bearing mice under different chemotherapeutic treatments. (n = 6 per group). Treated group (T) - Control group (C) = difference between median survival (days) of T *vs.* C (TGD). (T - C)/C (%TGD). The data are shown as mean ± s.d., * = p < 0.05, ** = p < 0.01, n.s. = p > 0.05 by one-way ANOVA test.

The antitumor efficacy of EVs-Dox was evaluated on the metastatic xenograft models of human ovarian cancer that was established in BALB/c nude mice by *i.p*. injection with 1 × 10^7^ SKOV3 cells, and the tumor growth was monitored using MRI. After seven days of tumor inoculation, solid portion (white arrows) was noninvasively detected as low signal in T1 weighted imaging (T1WI) but hyperintense signal in T2 weighted imaging (T2WI), representing tumor nodules which spread though the peritoneal cavity and metastasized to nearby organs (Figure 6A). This condition could be diagnosed as stage II ovarian cancer according to the American Joint Committee on Cancer Staging (AJCC)'s TNM classification of malignant tumors 35. Then tumor-bearing mice were treated with Dox-loading EV subsets as well as the positive control Lipo-Dox and DoxHCl every other day for two weeks with a total of seven doses (Figure 6B).* I.p.* injection was chosen as an administration route, since it had been widely used as a rational approach for advanced ovarian cancer therapy in clinic 36. After animal sacrifice on day 28, main organs including liver, spleen, uterine appendage, heart, kidneys and gastrointestinal tract were harvested from inoculated mice for gross and histopathological evaluation. We found that large amounts of tumor cells primarily invaded into gastrointestinal track (Figure S8, arrow heads), followed by liver, spleen, thoracic diaphragm, kidney and stomach (arrow heads, Figure 6C-6E and Figure S9), which was further confirmed by histological examinations using H&E staining (black dashed circles). The number of tumor nodules on organ surface (Figure 6F) further demonstrated that the MiV-Dox significantly suppressed tumor metastasis (3.8 ± 2.2 nodules/mouse) compared with ApB-Dox (14.7 ± 5.9 nodules/mouse) and Exo-Dox (17.5 ± 6.9 nodules/mouse). By contrast, the mice receiving commercial formulation Lipo-Dox was slightly superior to untreated mice. As previous studies reported 37, DoxHCl indeed induced severe cardiotoxicity featured by myofibrillar degeneration and myocytes vacuolization (Figure 6G) as well as a significant reduction in body weight (Figure 6H and Figure S10). Therefore, median survival time of the mice treated with DoxHCl was largely decreased in comparison with untreated mice. However, Dox-loaded in EVs as well as Lipo considerably improved the survival rate to varying degrees. In particular, survival time of the mice receiving MiV-Dox was prolonged beyond 160 days (Figure 6I).

**Figure 7:**
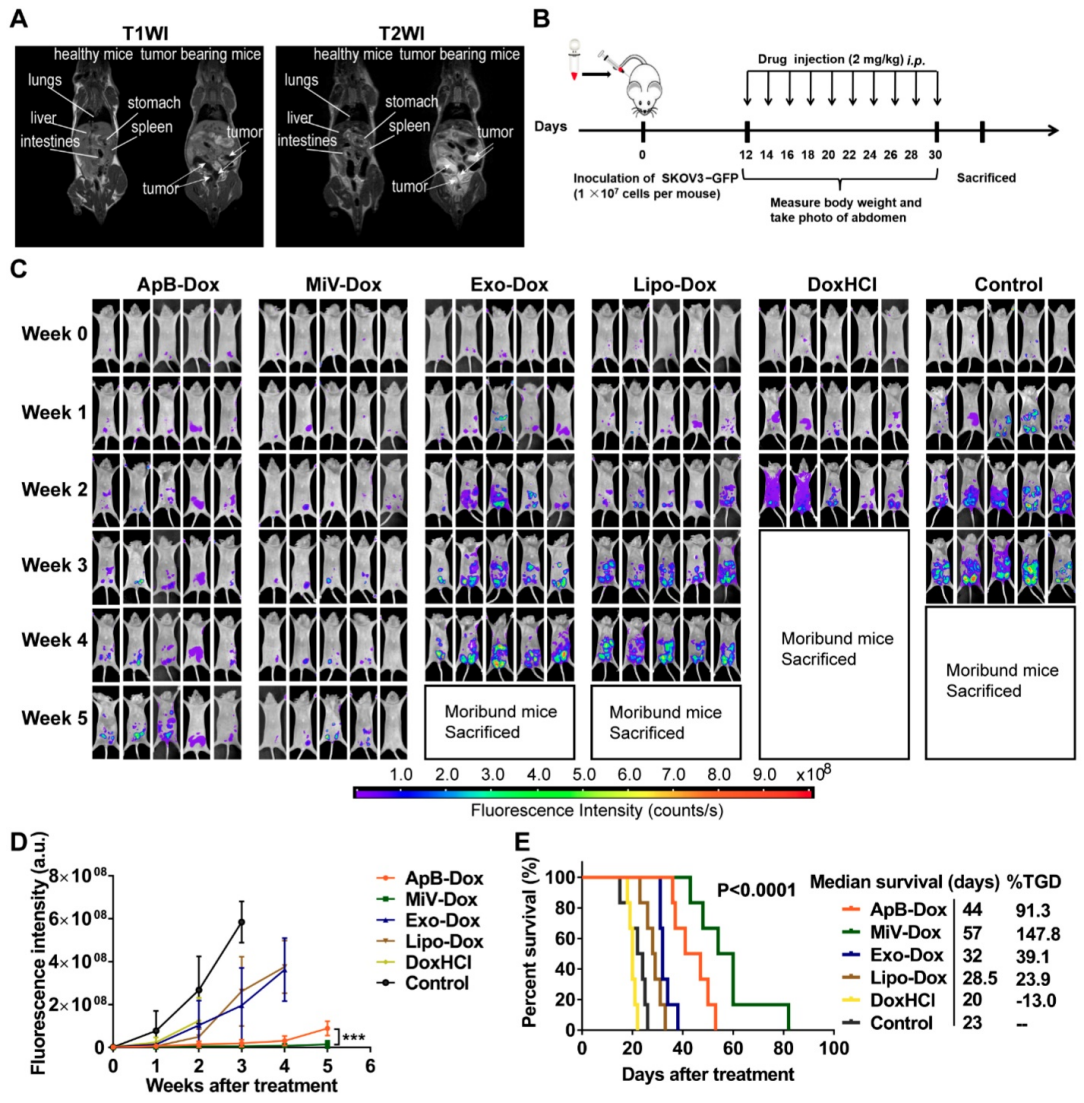
*In vivo* therapeutic efficacy of EVs-Dox against stage III ovarian carcinoma. (A) T1WI and T2WI of healthy mouse and tumor-bearing mouse in the coronal plane. Arrows indicate metastatic regions. (B) Experimental procedure of tumor induction and therapeutic regimen. (C) The images of GFP-expressing tumor-bearing mice during five-week-treatments of various formulations. At baseline (week 0) all groups showed equal abdomen fluorescence indicative of equal tumor burden. By week 1 to 5, the tumor burden was reduced in the mice treated with MiV-Dox compared with controls. (D) Quantitative fluorescence intensity of GFP in tumor-bearing mice with or without any treatments for 5 weeks. (E) Kaplan-Meier survival curves and the median survival time of mouse models of metastatic ovarian cancer after various treatments (n = 6 per group). Treated group (T) - Control group (C) = difference between median survival (days) of T *vs.* C (TGD). (T - C)/C (%TGD). The data are shown as mean ± s.d., *** = p < 0.001 by one-way ANOVA test.

Next, we sought to examine the therapeutic potential of EVs-Dox on the tumor-bearing mice with higher metastatic malignancy. After the establishment of ovarian cancer model for 12 days, it seemed that tumor cells metastasized to distant organs out of the pelvis, which was monitored by MRI and further staged as grade III ovarian cancer (Figure 7A). In this case, GFP stably-expressing SKOV3 cells were also employed to track tumor progression. Likewise, we then conducted *i.p.* injection with various Dox formulations at a dose of 2 mg/kg every two days (ten doses totally, Figure 7B). Impressively, tumor cells emitting GFP fluorescence were nearly eliminated in the mice treated with MiV-Dox, while ApB-Dox exhibited significantly lower therapeutic efficacy which was further confirmed by fluorescent intensity quantification (Figure 7C and 7D). Correspondingly, MiV-Dox improved survival relative to ApB-Dox, Exo-Dox and Lipo-Dox (Figure 7E). Together, these results further validated that, among the three types of EVs, MiV-Dox significantly inhibited tumor metastasis and improved survival *in vivo*, implying MiV-Dox is superior in tumor-selective delivery and accumulation.

## Discussion

In the past decade, EVs have gained increasing attention as potential carriers for site-specific drug delivery 38. However, the exact mechanisms underlying how EVs exhibit specific organ selectivity are not fully elucidated. The intrinsic targeting capability is probably endowed by secreting cells, since all EV compositions originate from them.

Our findings support this hypothesis that M1 macrophage-derived EVs indeed display the tumor tropism like their donor cells dependent on chemokine signaling pathway, since a CCR2 antagonist significantly decreased the tumor accumulation of EVs. Then it raises a question as to which EV subtypes equipped with CCR2 or other specific components are associated with targeting mechanism. Due to the heterogeneous EVs varying in size, contents and biogenesis, we sought to isolate the three main types of EVs (ApB, MiV and Exo) by differential centrifugation, which has been considered as the most appropriate method to obtain high-purity EV subsets 39. Consistent with previous literatures showing an overlap in EV size 40, we also found it difficult to discern EV subsets by size and structure, in particular after removal of large pellets by extension of low-speed centrifugation step. Large particles were deemed undesirable for the targeted drug delivery, since they could be rapidly cleared from circulation 41. Despite the fact that the definition of EV subsets generally relies on size, vesicles classification by biogenesis pathways is more likely to reflect the biological and functional relevance 42. In our study, diversity in protein, lipid and RNA profiles implicates each pellet identified as different EV subtypes originate from distinct biogenetic pathways. Notably, the purity of each EV subpopulations has been confirmed by specific EV markers TSP1, ARF6, ALIX and TSG101. MiV are the major EV subpopulation and packaged with the maximum protein compositions from M1 macrophages. Given that MiV are formed by directly shedding from plasma membrane 43, massive membrane transport proteins are detected in MiV, while the lipid contents closely resemble those of plasma membrane. Remarkably, M1 macrophage surface marker CCR2 is enriched in MiV, but barely detected in ApB and Exo. Since human CCL2 could bind to murine CCR2 44, we speculate MiV may harness CCL2/CCR2 chemokine axis to track tumor cells, which is similar to donor cells. Though MiV rank as the largest group in EVs, it is hard to preclude the possibility that ApB and Exo may be able to target tumor without CCR2 involvement. ApB derive from membrane blebbing in apoptotic cells, whereas Exo come from intracellular vesicles after fusion with plasma membrane. Therefore, ApB and Exo surface are inevitably incorporated with varying degrees of certain membrane compositions, which, in combination with CCR2 by M1 macrophages, may recognize and interact with target cells 45. Improvements in EV isolation techniques are urgently needed to discriminate target specificity among heterogeneous EV subpopulations in the future work. Meanwhile, there is another very interesting open question as to whether some artificially membrane vesicles produced by filter-extrusion of cells membrane may carry targeting moieties. Although this approach seems simple and feasible, it is difficult to control the purity and quality of vesicles compare with those produced from “nature factory”. In addition to parent cell source, administration route may also influence biodistribution profile of EVs. Considering that EVs highly localize in the liver and spleen but barely in tumor tissues after systemically injection 6, local injection seems to be an effective method for EV delivery to metastatic ovarian cancer cells which are mostly confined within the peritoneal cavity.

Once arriving at receipt cells, MiV are able to release drug cargo by SNARE-mediated membrane fusion machinery by which Dox rapidly accumulates in the nucleus leading to robust cell death without induction of cell invasion. By contrast, ApB-Dox and Exo-Dox display moderate anti-tumor activity and fail to prevent cell invasion, probably because a large proportion of them is trapped in endosomal degradative pathways post-uptake. Intriguingly, our data demonstrate that EVs as chemotherapeutic drug carriers significantly decrease the incidence of multidrug resistance compared to synthetic lipid-based Dox formulation (Lipo-Dox) and free drug (DoxHCl), which is similar to previous studies 7, 46. Drug resistance is the main cause of chemotherapy failure in ovarian cancer treatment leading to tumor reoccurrence and metastasis 47. As expected, MiV-Dox substantially suppressed tumor growth and metastasis in late-stage ovarian cancer murine models, as evidenced by an optimum tumor-specific formulation. Furthermore, this treatment markedly reduced Dox-induced cardiac dysfunction and consequently showed survival benefits. Notably, there is a statistically significant improvement (though moderate) in antitumor activity for MiV-Dox compared with ApB-Dox. Presumably, healthy cells and apoptotic cells integrated different cell components exerting distinct function into MiV and ApB, which warrants further investigations. In fact, the occurrence of apoptosis is typically a rare event; therefore, low yield of ApB may limit the further scale-up production. On the basis of our findings, however, Exo are unlikely to specifically deliver drug to tumor site, showing undesirable chemotherapy outcomes like Lipo.

## Conclusions

In this study, we managed to isolate and identify the three main subtypes of M1 macrophage-derived EVs: ApB, MiV and Exo, and revealed that MiV possess the superior advantageous features to other EV subtypes for chemotherapeutic drug delivery. Due to replication of donor cell membrane composition and function related with tumor tropism dependent on CCL2/CCR2 chemokine axis, MiV loaded with Dox are able to specifically recognize and interact with tumor cells and bypass phagocytic clearance, and then utilize the mechanism of SNARE-mediated membrane fusion to facilitate direct drug transport to nucleus rather than endocytic degradation. In addition to desirable therapeutic outcomes of MiV in advanced stage ovarian cancer, high yield, drug-payload and membrane stability guarantee MiV large-scale production and clinical translation, although further optimizations remain to be implemented.

## Abbreviations

EVs: extracellular vesicles
ApB: apoptotic bodies
MiV: microvesicles
Exo: exosomes
Lipo: liposomes
CCL2: monocyte chemoattractant protein-1
CCR2: C-C chemokine receptor type 2
TSP1: thrombospondin-1
ARF6: ADP-ribosylation factor 6
TSG101: tumor susceptibility gene 101
ALIX: apoptosis linked gene-2-interacting protein X
LPS: lipopolysaccharide
IFN-γ: interferon γ
NTA: nanoparticle track analysis
tRPS: tunable resistive pulse sensing method
DoxHCl: doxorubicin hydrochloride
MDR1: multidrug resistance protein 1
LC-MS/MS: liquid chromatography-tandem mass spectrometry
DAPI: 4′, 6-Diamidino-2-phenylindole dihydrochloride
DMSO: dimethyl sulfoxide
MTT: 3-(4,5-dimethylthiazol-2-yl)-2,5-diphenyl tetrazolium bromide
GFP: green fluorescent protein
Lipo-Dox: lipid-based Dox formulation
MRI: magnetic resonance imaging
TEM: transmission electron microscope
SEM: scanning electron microscope
RTCA: real time cell analyzer
