# Supplementary material for: Proinflammatory macrophage-derived microvesicles exhibit tumor tropism dependent on CCL2/CCR2 signaling axis and promote drug delivery via SNARE-mediated membrane fusion
Source: Theranostics. 2020 May 17;10(15):6581–98. doi: 10.7150/thno.45528 (PMC7295053; doi:10.7150/thno.45528)
Supplement: Supplementary file 1 — Supplementary figures and tables. [file thnov10p6581s1.pdf]

Proinflammatory macrophage-derived microvesicles exhibit tumor tropism dependent on CCL2/CCR2 signaling axis and promote drug delivery *via* SNARE-mediated membrane fusion

Ling Guo[1,2], Ye Zhang[1,2], Runxiu Wei[1,2], Xiaochen Zhang[1,2], Cuifeng Wang[1,2], Min Feng*[1,2]*

[1]School of Pharmaceutical Sciences, Sun Yat-sen University, University Town, Guangzhou, 510006, P.R. China

[2]Guangdong Provincial Key Laboratory of Chiral Molecule and Drug Discovery, School of Pharmaceutical Science, Sun Yat-sen University, Guangzhou, 510006, P.R. China

*Corresponding authors: Tel.: +8620 39943073; fax: +8620 39943073

Email address: wangcf6@mail.sysu.edu.cn and fengmin@mail.sysu.edu.cn

SUPPLEMENTARY FIGURES

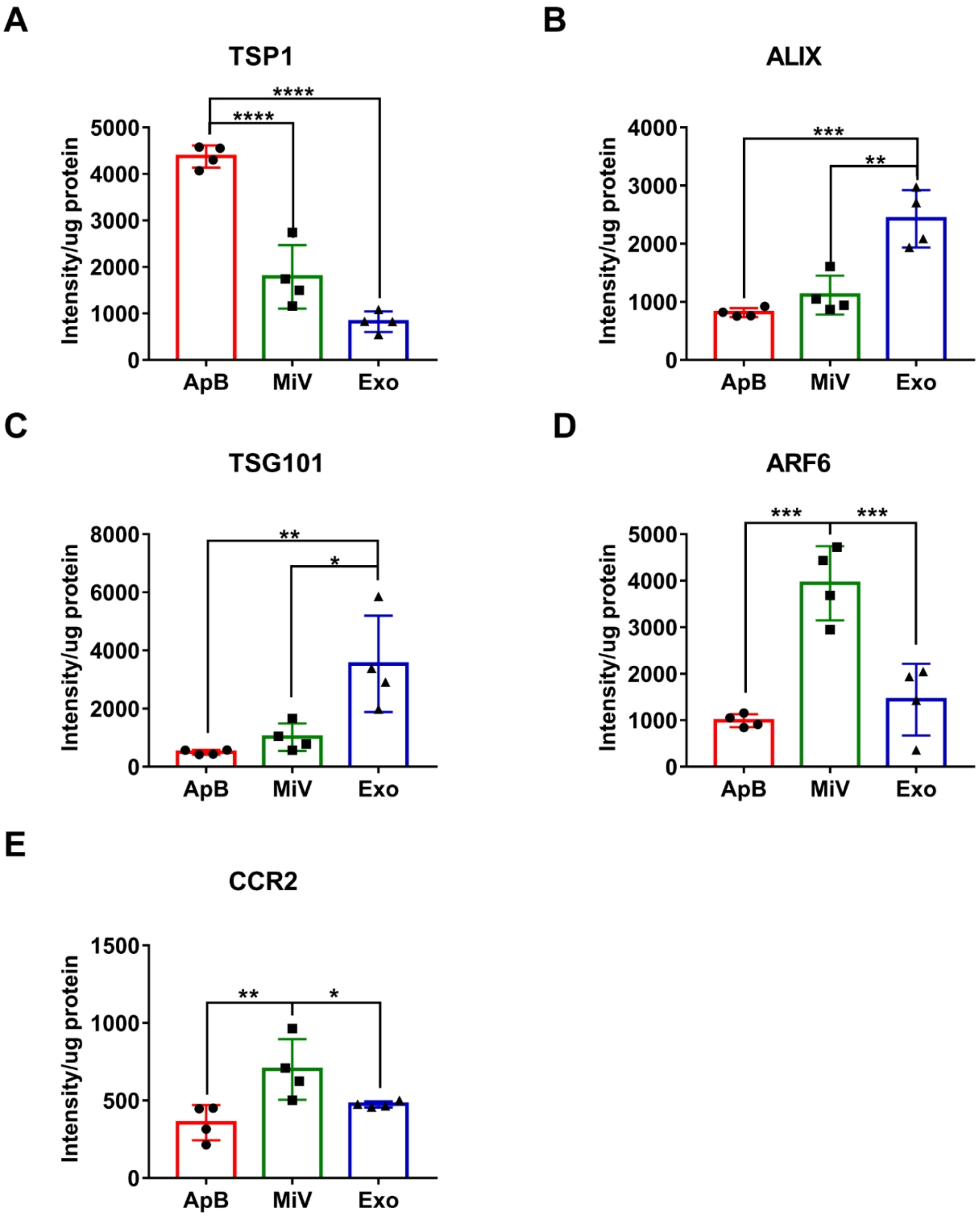

Figure S1. (A-E) Quantitative protein expression levels of EV subtypes by western blotting analysis (n = 4). The data were shown as mean ± s.d., * was $p < 0.05$, ** was $p < 0.01$, *** was $p < 0.001$, **** was $p < 0.0001$ by one-way ANOVA test.

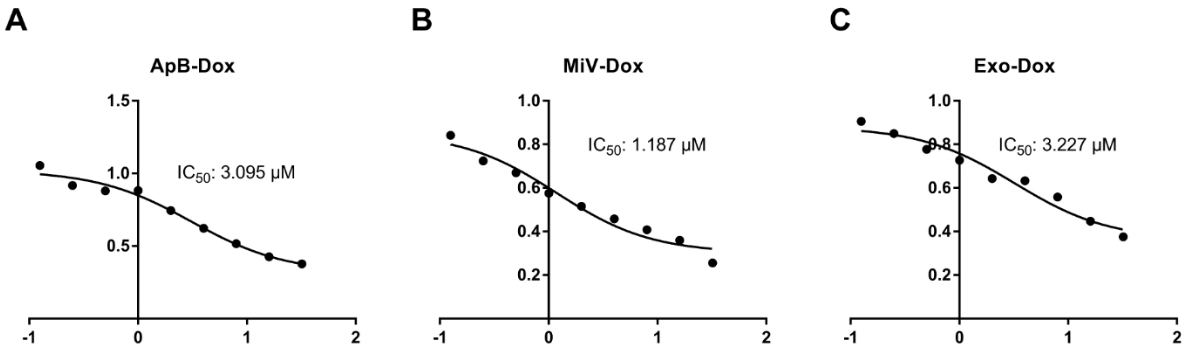

Figure S2. The IC_{50} value of ApB-Dox, MiV-Dox and Exo-Dox calculated by the GraphPad Prism7 software.

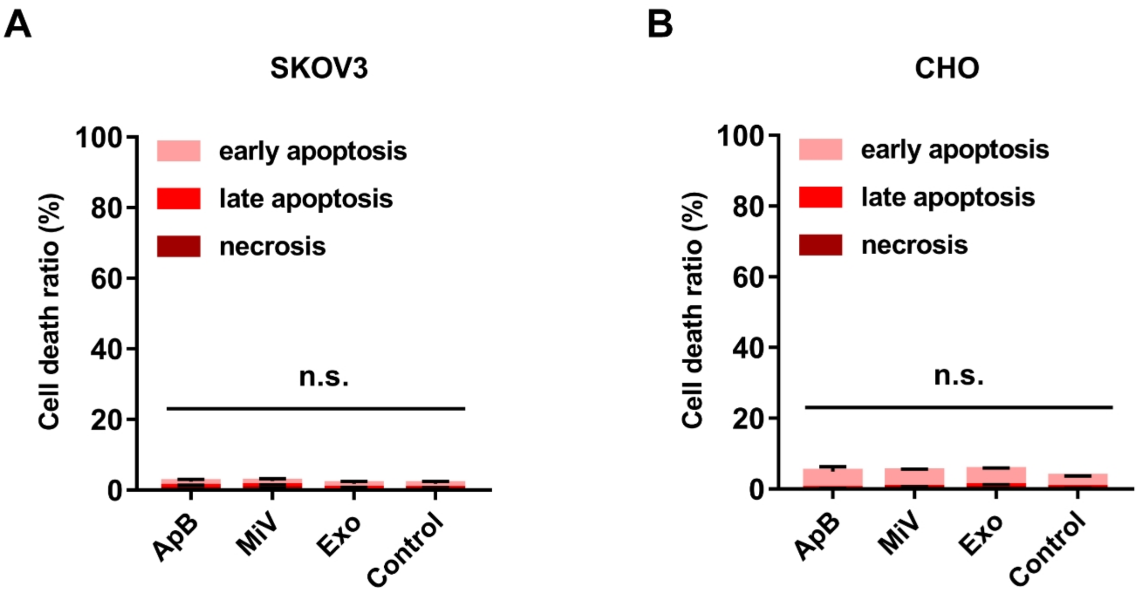

Figure S3. Annexin V-FITC/PI assay for apoptosis detection of SKOV3 cells (A) and CHO cells (B) under the treatment of drug-free EVs for 24 h. Quantitative data showed the percentage of early apoptosis, late apoptosis and necrosis cells. The data were shown as mean ± s.d., n.s. was $p > 0.05$ by one-way ANOVA test.

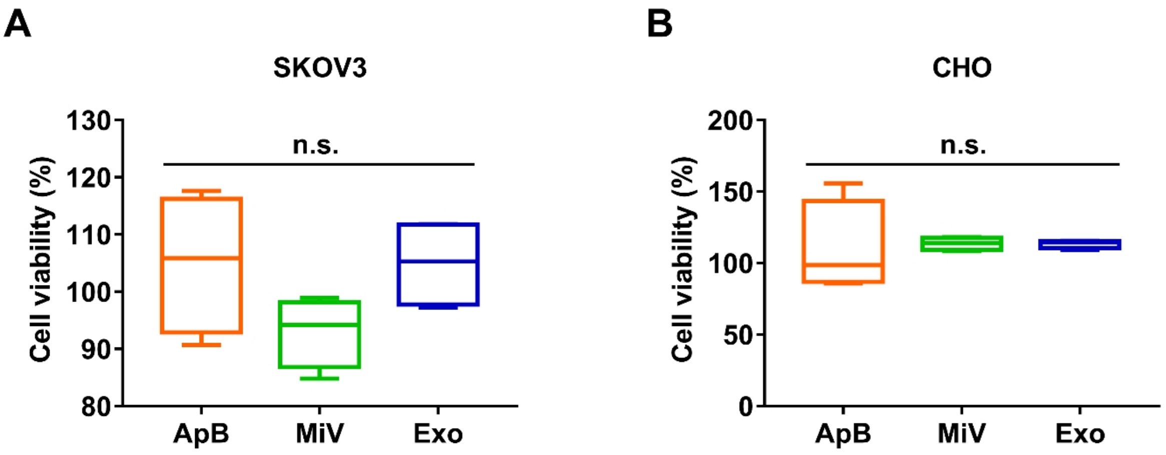

Figure S4. Cell viability of SKOV3 cells (A) and CHO cells (B) treated with drug-free EVs for 24 h. The data were shown as mean ± s.d., n.s. was $p > 0.05$ by one-way ANOVA test.

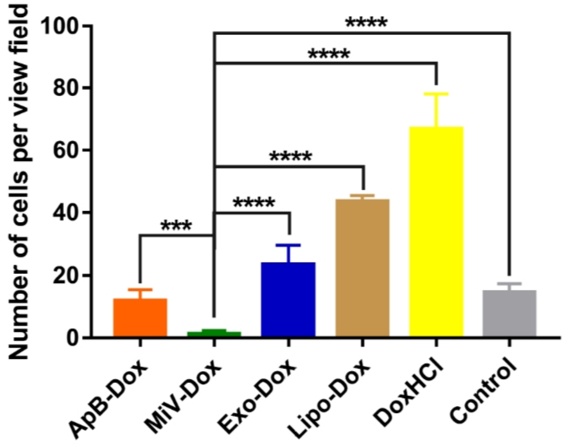

Figure S5. Quantification of the number of live cells per each view field from corresponding fluorescence images present in Figure 4I ($n = 11$). The data were shown as mean ± s.d., *** was $p < 0.001$, **** was $p < 0.0001$ by one-way ANOVA test.

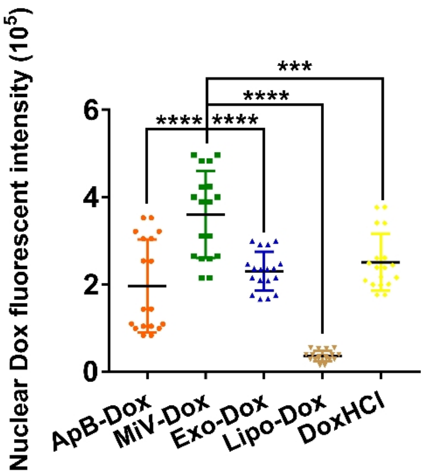

Figure S6. The Dox fluorescence signal in SKOV3 nuclei was quantificationally analyzed by ImageJ software ($n = 20$). The data were shown as mean ± s.d., *** was $p < 0.001$, **** was $p < 0.0001$ by one-way ANOVA test.

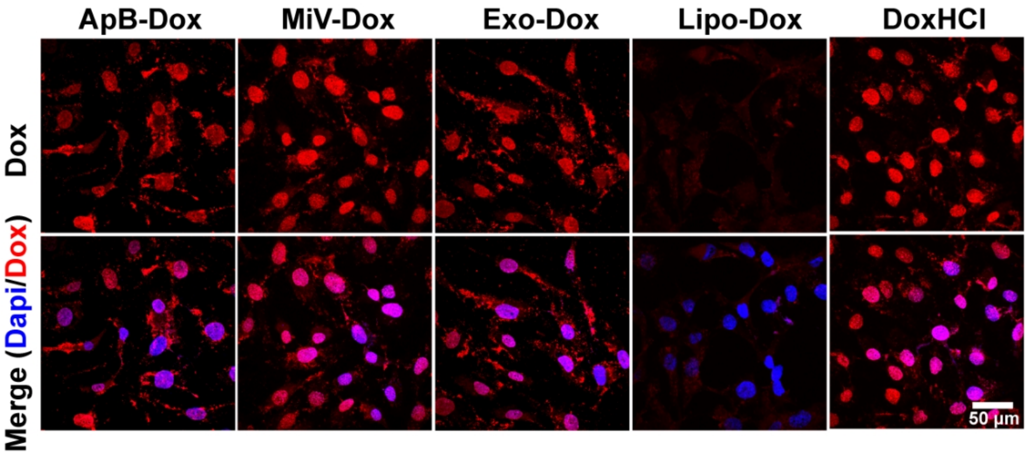

Figure S7. Confocal laser scanning microscopy images showed the intracellular distribution of Dox of SKOV3 cells after treated with various formulations at 4 h. The nuclei were stained with DAPI (blue). Dox produced the red fluorescence. The merged images were the overlay of two individual images. Scale bar was 50 μm.

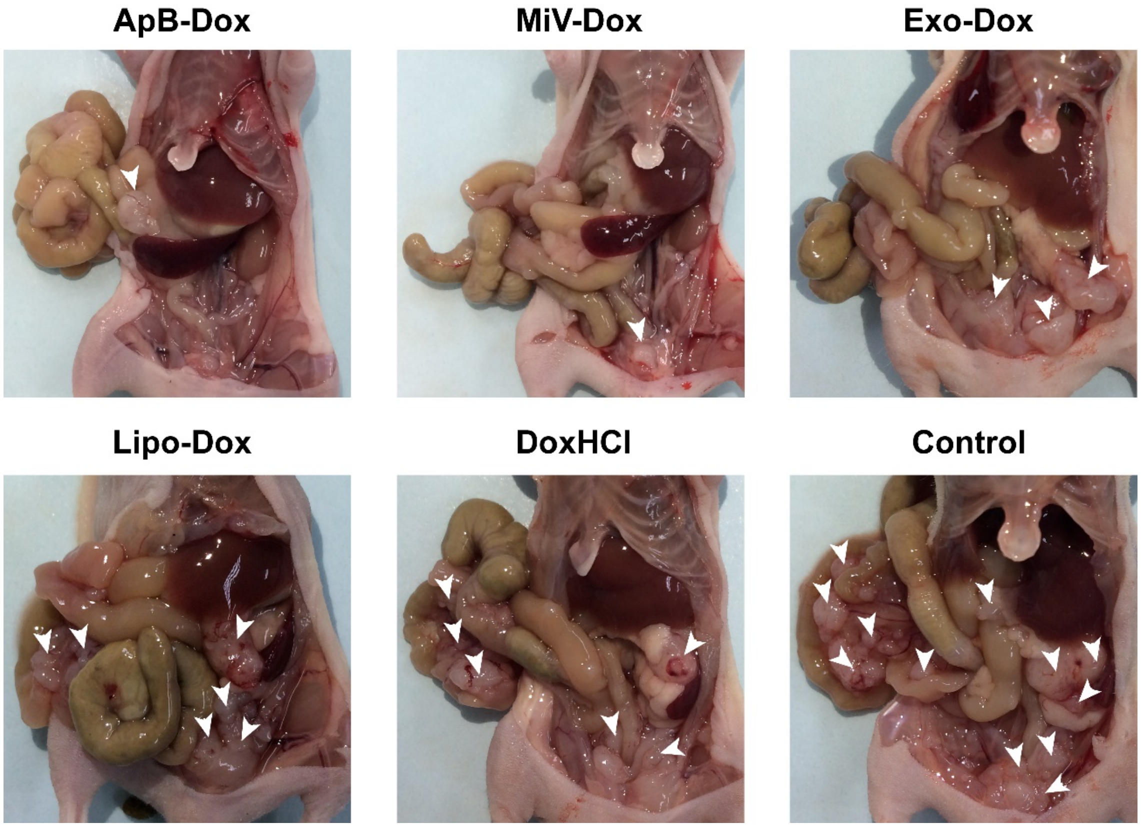

Figure S8. Representative microscopic images of peritoneal cavity in mice from six experimental groups on the 28th treatment day. White arrow head indicated tumor nodules.

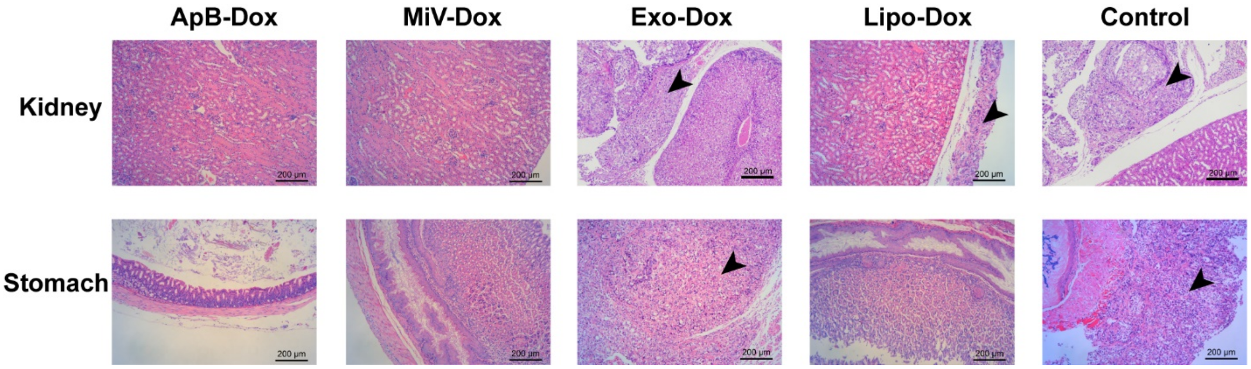

Figure S9. H&E stained kidneys and stomachs from control and treated tumor-bearing mice on day 28. Tumor nodules were indicated by arrow heads. Scales bar were shown in each image.

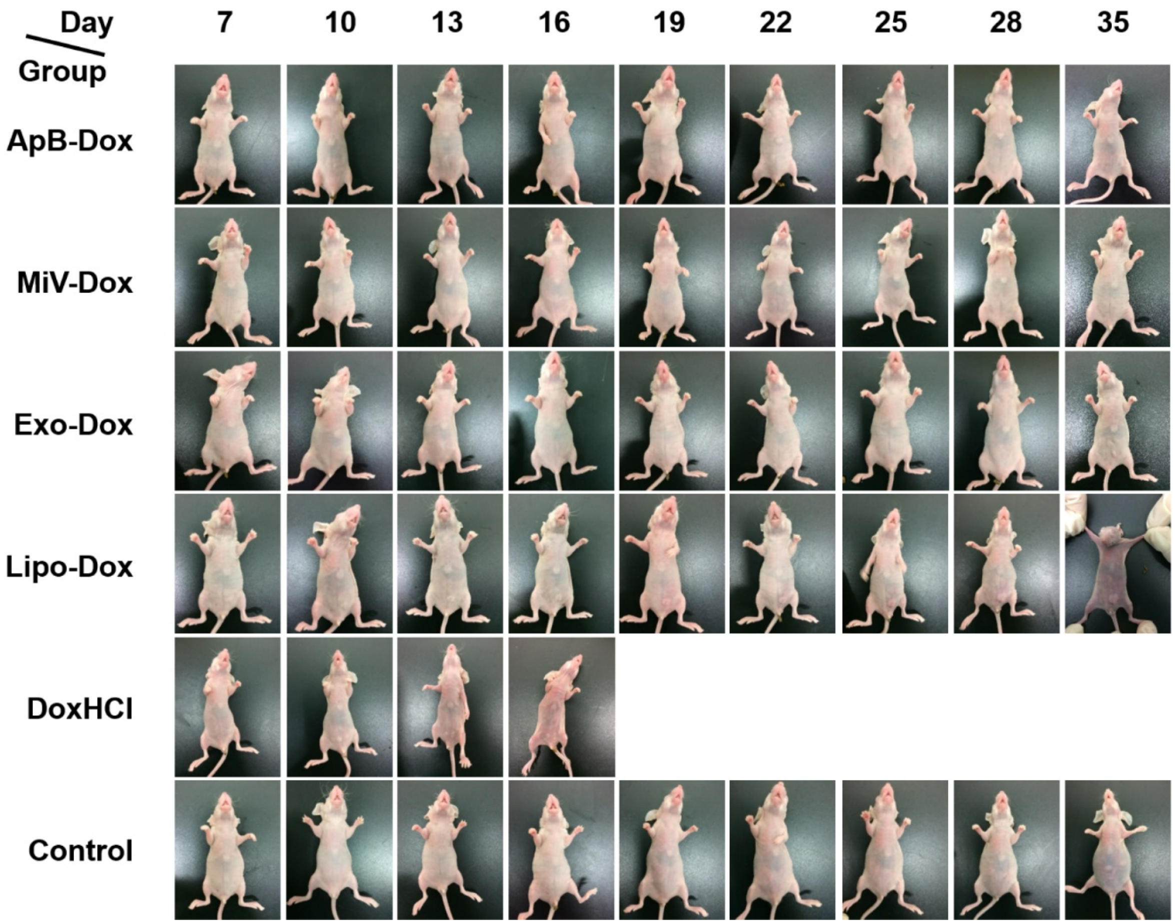

Figure S10. *In vivo* safety and toxicity evaluation in mice. 1×10^7 SKOV3 cells were injected into the abdomen of the BALB/c nude mice followed by serial imaging studies in 3-days intervals from day 7.

Table S1. Encapsulation efficiency (EE) of Dox in each EV subtype.

	ApB	MiV	Exo
EE (%)	96.70 ± 0.38	97.69 ± 0.91	96.95 ± 1.47
